# Safety and efficiency of ventricular pacing prevention with an AAI-DDD changeover mode in patients with sinus node disease or atrioventricular block: impact on battery longevity—a sub-study of the ANSWER trial

**DOI:** 10.1093/europace/euv358

**Published:** 2015-11-26

**Authors:** Martin Stockburger, Pascal Defaye, Serge Boveda, Branislav Stancak, Arnaud Lazarus, Johann Sipötz, Stefano Nardi, Mara Rolando, Javier Moreno

**Affiliations:** 1 Department of Cardiology and Angiology, Charité University Hospital, Berlin, Germany; 2 Department of Cardiology, Havelland Kliniken Nauen, Ketziner Strasse 21, 14641 Nauen, Germany; 3 Arrhythmia Unit, Cardiology Department, University Hospital, Grenoble, France; 4 Arrhythmia Unit, Clinique Pasteur, Toulouse, France; 5 Arrhythmia Department, Hospital of Eastern Slovakia, Institute of Cardiovascular Diseases, Kosice, Slovakia; 6 InParys, A. Paré Private Hospital, Neuilly sur Seine, France; 7 Hanusch Krankenhaus, Vienna, Austria; 8 Azienda Ospedaliera S. Maria, Terni, Italy; 9 Sorin Group International SA, Lausanne, Switzerland; 10 Arrhythmia Department, Cardiovascular Institute, San Carlos University Hospital, Madrid, Spain; 11 Arrhythmia Department, Hospital Ramón y Cajal, Madrid, Spain

**Keywords:** SafeR, Pacing prevention, Atrioventricular block, Sinus node dysfunction, Pacemaker mode selection, Battery impedance, Device longevity, Replacement

## Abstract

**Aims:**

This ANSWER (Evalu*A*tio*N* of the *S*afeR mode in patients *W*ith a dual chamb*ER* pacemaker indication) sub-study assesses safety and effectiveness of SafeR™ and the impact of ventricular pacing (VP) prevention on anticipated device longevity and replacement rate.

**Methods and results:**

Patients implanted for atrioventricular block (AVB, *n* = 310) or sinus node dysfunction (SND, *n* = 336) were randomly assigned to SafeR (*n* = 314) or DDD (*n* = 318) and followed for 36 months. Safety, median VP, estimated device longevity (mean difference, 95% confidence interval [CI]), and anticipated replacement rates were analysed by pacing mode and implant indication. No difference in mortality, syncope, or mode intolerance was observed between randomization groups regardless of the indication. Ventricular pacing on SafeR vs. DDD was 11.5 vs. 93.6% in the overall population (*P* < 0.001), 89.2 vs. 83.8% in permanent AVB (*P* = 0.944), 53.5 vs. 98.2% in intermittent AVB (*P* < 0.001), and 2.2 vs. 84.7% in SND (*P* < 0.001). Anticipated median device longevity increased on SafeR by 14 [Q1 10; Q3 17] months [10; 17] (*P* < 0.001) in the overall population, 9 months [−5; 22] (*P* = 0.193) in permanent AVB, 14 months [8; 19] (*P* < 0.001) in intermittent AVB, and 14 months [9; 19] (*P* < 0.001) in SND. In intermittent AVB and SND, prolonged estimated battery longevity translated into the prevention of one anticipated replacement in at least 23% of patients.

**Conclusion:**

SafeR was effective in reducing VP in intermittent AVB and in SND. No effect was observed in permanent AVB. No safety issue was observed. Ventricular pacing reduction by SafeR translated into relevant estimated prolongation of device longevity and anticipated reduction of required replacements.

**What's new?**

- For the first time, our study reports safety and effectiveness of a mode for ventricular pacing (VP) minimization, namely SafeR™, in patients implanted for intermittent and permanent atrioventricular block (AVB), compared with standard DDD.
- No difference in mortality, syncope, or pacing mode intolerance was reported with SafeR™ vs. DDD, in each pacing indication.
- SafeR was effective in reducing VP in intermittent AVB as well as in sinus node dysfunction patients. Ventricular pacing reduction translated into relevant prolongation of estimated device longevity and diminished anticipated need for replacement.

## Introduction

During the last two decades, electromechanical cardiac dyssynchrony has been recognized as a crucial adverse factor that causes and aggravates heart failure (HF). Cardiac dyssynchrony can be provoked by spontaneously delayed conduction^1^ or by right ventricular (RV) pacing.^2^ Several retrospective^3^ and prospective^4,5^ studies have demonstrated the potential of RV pacing to promote HF symptoms and atrial fibrillation (AF). Accordingly, intense efforts have been made to reduce as far as possible unnecessary ventricular pacing (VP) even when delivered in the atrioventricular (AV) sequential pacing mode (DDD). In this context, the AAI(R) mode has long been considered the most appropriate pacing mode for the majority of patients suffering from sinus node dysfunction (SND). In the period that followed, refined technical algorithms, such as AAI-to-DDD changeover modes like Managed Ventricular Pacing (MVP™)^6^ or AV search hysteresis modes, have been developed and assessed, mainly in SND patients. It led to the introduction in the ESC guidelines of a recommendation to avoid unnecessary VP in SND patients, while the use of these algorithms remains controversial in patients with impaired AV conduction due to the lack of clinical results in these populations of patients.^7^

The SafeR AAI-DDD changeover mode (Sorin CRM SAS, Clamart, France) was developed in accordance with these objectives and has been successfully used in clinical practice for several years.^8,9^ The results of the ANSWER randomized trial (Evalu*A*tio*N* of the *S*afeR mode in patients *W*ith a dual chamb*ER* pacemaker indication, ClinicalTrials.gov Identifier: NCT00562107) have recently been published.^10^ This trial shows that the use of SafeR to reduce VP in a general dual-chamber pacemaker population, comprising similarly sized subgroups of SND and atrioventricular block (AVB) patients, is effective and safe. The clinical co-primary endpoint of this study (hospitalization for HF or AF or cardioversion) did not differ between groups, however. Secondary endpoint analyses nevertheless supported the hypothesis that SafeR vs. DDD may prevent HF events. In line with the ANSWER primary endpoint results, the Danish pacemaker trial (DANPACE) and the recently published MINimizE Right Ventricular pacing to prevent Atrial fibrillation and heart failure (MINERVA) study showed in patients with SND and patients with paroxysmal AF no benefit by VP prevention in terms of mortality or a combination of mortality, HF, or permanent AF. This sub-analysis of the ANSWER study further investigates in a mixed pacemaker patient population, but with a particular focus on AVB patients, safety, and effectiveness of VP prevention with SafeR. In addition, we evaluate the importance of VP reduction to prolong the expected battery longevity and to avoid anticipated pulse generator exchanges, as extended device longevity could certainly be seen as an advantage of VP prevention beyond the aforementioned clinical endpoints.

## Methods

### Study design

The ANSWER study design has already been published.^10^ In brief, the ANSWER study was an investigator-initiated, prospective, randomized, single-blind, controlled, parallel-design, European multicentre (43 centres) trial. Patients aged ≥18 years were included if they had a pacemaker indication based on applicable guidelines^7^ and had received a dual-chamber pacemaker equipped with the SafeR mode less than a month prior to enrolment. The pacemaker indication was based on the diagnosis of third-degree intermittent or permanent AVB and second-degree intermittent AVB or SND. Patients were excluded if they had permanent AF, sustained ventricular arrhythmias, congenital complete heart block, or vasovagal syncope. The study was conducted in accordance with the Declaration of Helsinki and Good Clinical Practice. The protocol was approved by the local ethics committees.

### SafeR™ pacing mode

All pacemakers used in the study were SafeR™ enabled. This algorithm provides AAI(R) pacing while continuously monitoring AV conduction, and ensures DDD(R) pacing when needed for all types of AVB. Switching to the DDD(R) mode occurs after detection of two consecutive non-conducted beats (third-degree AVB criterion), missing AV conduction in 3 of 12 atrial beats (second-degree AVB criterion), or ventricular asystole (regardless of the underlying rhythm, asystole interval programmable to ≥2 to 3 s). In addition, SafeR does not allow more than six consecutive intervals (between atrial and ventricular events) longer than a programmable value (ranging from 200 to 450 ms). Upon the seventh long PR interval, the SafeR algorithm switches to DDD (first-degree AVB criterion), in order to prevent AV decoupling that has been described as an undesired side effect of a different AAI-DDD changeover mode.^11,12^

The switch back to AAI occurs after 100 beats or 12 consecutive cycles with spontaneous R waves. The SafeR pacing mode also counts all detected AVB episodes and provides an accurate categorization of the kind of AVB.^13^ A more comprehensive description of the SafeR pacing mode can be found elsewhere.^10,14^

### Follow-up and data collection

After implant and enrolment, all pacemakers were programmed to the SafeR pacing mode for 1 month to allow equilibration of medical treatment. At the end of this run-in period, patients were randomized in a 1 : 1 fashion to either the SafeR pacing mode (SafeR group) or the conventional DDD(R) pacing mode (control group). In the control group, nominal AV delays were proposed as preferred setting (155 ms after a sensed atrial event and 220 ms after atrial pacing, with automatic shortening of the delay during a rate increase), but the programming was left to physician's discretion.

Follow-up visits were carried out after enrolment before hospital discharge, at 1 month (randomization visit), 6, 12, 18, 24, and 36 months. The medication and dosages were emphasized to be prescribed as medically indicated.

The Study Steering Committee blindly reviewed and analysed all serious adverse events for relationship with the device and pacing modes.

At each visit, the device memory was downloaded. Clinical data were retrieved and transferred to case report forms (CRFs). Adverse events (AEs) prompted unscheduled visits and were recorded on specific CRFs.

### Objectives and endpoints

ANSWER had a two-fold primary endpoint: a primary technical efficacy endpoint, the cumulative amount of VP at 1 year, and a primary clinical endpoint, a composite of hospitalization for HF, AF, or cardioversion at 3 years. Final results of the two-fold primary endpoint have recently been published.^11^

Secondary endpoints include the cumulative amount of VP at 3 years and AE related to the pacing system or pacing mode, globally and per implant indication. For this analysis, the primary pacemaker indications were categorized as permanent AVB, defined as third-degree AVB diagnosed as permanent, intermittent AVB (any other AV block), or SND.

Ancillary endpoints include device battery impedance at 3 years and estimated total longevities per randomization arm, estimated device longevities per randomization arm with preset programmed parameters, and the anticipated number of device replacements per randomization arm. Device battery impedance was retrieved from device memories at the last follow-up and estimated total longevity was determined by the programming software per randomization arm at the last follow-up as a function of pacemaker parameter settings, mean atrial and VP, lead impedances, and patient heart rate retrieved at this follow-up. Estimated total longevity at preset programmed parameters was calculated according to the pacing mode and the implant indication, at fixed pacing amplitudes and widths in both cavities (2.5 V and 0.35 ms, respectively), with rate response OFF and lead impedances approximated to 500 Ω. Anticipated device replacements were calculated using a Microsoft Excel^®^-based tool. Inputs into the model included device longevity and patents' survival probability. These parameters were integrated applying a computational approach that permitted the calculation of the anticipated percentage of patients experiencing a reduction of at least one replacement on SafeR vs. DDD.

### Sample size and statistical methods

The sample size calculation, based on the two-fold primary endpoint, led to a required sample size of 640 patients in total.

Secondary and ancillary endpoint analyses were carried out on the intention-to-treat population. Individuals with missing 3-year data for VP were included in the analysis using the Last Observation Carried Forward (LOCF) imputation method.

Quantitative or continuous parameters are presented as mean ± SD when the distribution followed a normal distribution and as median (Quartile I; Quartile III), if not. Mean difference (MD) and 95% confidence intervals (CI) were determined for device longevity. Qualitative parameters are presented descriptively as numbers and percentages.

The statistical analysis was performed using Student's *t*-test or the Wilcoxon rank test for quantitative parameters and *χ*^2^ or exact tests for qualitative parameters.

All statistical analyses were performed with the SAS^®^ statistical software, version 9.2.

## Results

### Study population

A total of 650 patients were included in the ANSWER study. Of those, 632 were randomized, 314 to the SafeR group and 318 to the DDD group. Eighteen patients were withdrawn before randomization due to death (2), implantation with a device other than Sorin Reply DR or Symphony DR (1), and consent withdrawal (6); two patients were lost to follow-up and seven presented with a major protocol deviation. Patient characteristics are summarized in *Table 1*. No significant difference was observed between the groups.

**Table 1:** Baseline characteristics of enrolled and randomized patients

	Included (*N* = 650)	SafeR group (*N* = 314)	DDD group (*N* = 318)
Age years, mean ± SD	72.4 ± 11.2	71.8 ± 12.2	72.9 ± 9.8
Male gender, *n* (%)	358 (55.2)	182 (58.0)	169 (53.1)
NYHA class, mean ± SD	1.6 ± 0.6	1.6 ± 0.6	1.6 ± 0.6
LVEF %, mean ± SD	58.3 ± 8.7	58.6 ± 9.1	58.2 ± 8.3
PR interval^a^ (ms), mean ± SD	191 ± 45	190 ± 47	192 ± 44
Indications for implant, *n* (%)			
Sinus node disease	336 (52.0)	167 (53.5)	160 (50.5)
AV block	310 (48.0)	145 (46.5)	157 (49.5)
Intermittent	270 (41.8)	127 (40.7)	136 (42.9)
Permanent	40 (6.2)	18 (5.8)	21 (6.6)
Other conduction disorders, *n* (%)	176 (29.2)	84 (28.7)	87 (29.6)
LBBB	64 (11.1)	29 (10.4)	31 (11.1)
LAFB	52 (8.7)	29 (9.9)	23 (7.9)
LPFB	1 (0.2)	1 (0.4)	0 (0.0)
Other	110 (17.9)	47 (16.0)	61(20.7)
History of arrhythmias disorders, *n* (%)	256 (39.4)	117 (37.3)	131 (41.2)
A	248 (38.2)	116 (37.1)	125 (39.3)
V	16 (2.5)	2 (0.6)	13 (4.1)
Underlying cardiac disease, *n* (%)	303 (46.6)	144 (45.9)	150 (47.2)
Coronary disease	183 (28.2)	87 (27.7)	91 (28.6)
Cardiomyopathy	31 (4.8)	15 (4.8)	13 (4.1)
Valvular disease	84 (12.9)	37 (11.8)	44 (13.8)
Associated conditions, *n* (%)	539 (82.9)	259 (82.5)	264 (83.0)
Heart failure	27 (4.2)	7 (2.2)	17 (5.3)
Diabetes	140 (21.5)	68 (21.7)	68 (21.4)
Angina	16 (2.5)	8 (2.5)	7 (2.2)
Pulmonary illness	32 (4.9)	13 (4.1)	17 (5.3)
Arterial hypertension	423 (65.1)	197 (62.7)	215 (67.6)
Other	252 (38.8)	121 (38.5)	124 (39.0)
Programmed parameters			
Rate response (%), OFF/LEARN/ON	17.0/58.2/24.8	18.1/60.0/21.9	15.6/57.3/27.1
Atrial output (V), median [Q1; Q3]	2.5 [2.5; 3.5]	2.5 [2.5; 3.5]	2.5 [2.5; 3.5]
Atrial width (ms), median [Q1; Q3]	0.35 [0.35; 0.35]	0.35 [0.35; 0.35]	0.35 [0.35; 0.35]
Ventricular output (V), median [Q1; Q3]	2.5 [2.5; 3.5]	2.5 [2.5; 3.5]	2.5 [2.5; 3.5]
Ventricular width (ms), median [Q1; Q3]	0.35 [0.35; 0.35]	0.35 [0.35; 0.35]	0.35 [0.35; 0.35]
A lead impedance (Ω), mean ± SD	461.1 ± 122.4	460.1 ± 122.9	462.8 ± 122.0
V lead impedance (Ω), mean ± SD	546.7 ± 157.2	539.3 ± 151.5	554.7 ± 162.3

LAFB, left anterior fascicular block; LBBB, left bundle block branch; LEARN, programming mode of the rate response functionality that enables the calibration of the activity sensors; LPFB, left posterior fascicular block; LVEF, left ventricular ejection fraction; NYHA, New York Heart Association; RBBB, right bundle block branch; AV, atrioventricular; A, atrial; V, ventricular.

^a^Only in SND or first-degree AV block.

During the 3-year follow-up, 31 patients randomized to SafeR and 51 to DDD underwent a change of programming: 23 patients were reprogrammed from SafeR to DDD and 8 from SafeR to VVI; 38 patients were reprogrammed from DDD to SafeR and 13 from DDD to VVI.

The mean follow-up was 918.7 ± 341.6 days. Five hundred and fifty-eight patients participated in the analysis of VP, and in 163 out of them, the last observation had to be carried forward. Six hundred and two patients participated in the analysis of device battery impedance, and 624 patients were included in the analysis of estimated device longevity.

### Cumulative ventricular pacing globally and per implant indications

At 3-year follow-up, the cumulative prevalence of VP (median [Q1; Q3]) remained significantly lower in the SafeR group (11.5% [0.1%; 73.8%]) than in the DDD group (93.6% [62.3%; 99.2%]), with an MD of −41% (CI: −47 to –35%; *P* < 0.001). This significant difference was also observed in the sub-populations of patients with AVB and SND (MD: −32%, CI: −40 to −23%, *P* < 0.001 and MD: −47%, CI: −55 to −40%, *P* < 0.001, respectively), though the percentage of VP was increased among patients with AV block on both treatments (*Table 2*). Patients with intermittent AVB experienced a significant decrease in VP with SafeR (MD: −36%, CI: −44 to −28%, *P* < 0.001), whereas those with permanent AVB had similar VP on SafeR and DDD (MD: −4%, CI: −31 to 23%, *P* = 0.944; *Table 2*).

**Table 2:** VP at 3 years, device battery impedance at 3 years, and estimated total device longevity according to implant indications in each pacing mode

Implant indication	AVB all (*N* = 297)			AVB perm (*N* = 39)			AVB int (*N* = 258)			SND (*N* = 325)		
Pacing mode	SafeR	DDD	*P*-value	SafeR	DDD	*P*-value	SafeR	DDD	*P*-value	SafeR	DDD	*P*-value
RR OFF/LEARN/ON (%)	21/67/12	19/65/16	NS	11/72/17	14/71/14	NS	22/66/11	19/64/16	NS	16/54/31	13/49/38	NS
A output (V)^a^	2.5 [2.5; 3.5]	2.5 [2.5; 3.5]		2.5 [2.5; 3.5]	2.5 [2.5; 3.5]		2.5 [2.5; 3.5]	2.5 [2.5; 3.5]		2.5 [2.5; 3.5]	2.5 [2.5; 3.5]	
A width (ms)^a^	0.35 [0.35; 0.35]	0.35 [0.35; 0.35]		0.35 [0.35; 0.35]	0.35 [0.35; 0.35]		0.35 [0.35; 0.35]	0.35 [0.35; 0.35]		0.35 [0.35; 0.35]	0.35 [0.35; 0.35]	
V output (V)^a^	2.5 [2.5; 3.5]	2.5 [2.5; 3.5]		2.5 [2.5; 3.5]	2.5 [2.5; 3.5]		2.5 [2.5; 3.5]	2.5 [2.5; 3.5]		2.5 [2.5; 3.5]	2.5 [2.5; 3.5]	
V width (ms)^a^	0.35 [0.35; 0.35]	0.35 [0.35; 0.35]		0.35 [0.35; 0.35]	0.35 [0.35; 0.35]		0.35 [0.35; 0.35]	0.35 [0.35; 0.35]		0.35 [0.35; 0.35]	0.35 [0.35; 0.35]	
A lead Z (Ω)^b^	447 ± 115	442 ± 106		472 ± 143	450 ± 94		443 ± 110	441 ± 107		473 ± 129	487 ± 135	
V lead Z (Ω)^b^	529 ± 168	539 ± 155		496 ± 111	523 ± 98		534 ± 174	541 ± 163		548 ± 135	573 ± 168	
VP at 3 years (%)^a^	55.0 [1.7; 97.1]	97.9 [81.0; 99.6]	<0.001	89.2 [24.0; 99.3]	83.8 [24.0; 98.8]	0.944	53.5 [1.7; 95.9]	98.2 [83.4; 99.6]	<0.001	2.2 [0.0; 29.9]	84.7 [32.8; 97.7]	<0.001
AP at 3 years (%)^a^	28.9 [9.8; 54.8]	28.1 [8.4; 65.0]	0.670	21.8 [8.0; 37.9]	43.7 [2.6; 73.5]	0.304	29.1 [10.5; 55.4]	27.5 [9.0; 64.5]	0.979	65.0 [33.1; 88.3]	70.1 [30.5; 92.0]	0.662
Battery impedance at 3 years (kΩ)^a^	0.46 [0.32; 0.59]	0.55 [0.34; 0.68]	0.009	0.44 [0.21; 0.59]	0.47 [0.36; 0.66]	0.482	0.46 [0.32; 0.57]	0.56 [0.34; 0.68]	0.011	0.44 [0.29; 0.60]	0.51 [0.32; 0.66]	0.066
Estimated device longevity (months)^b^	129.6 ± 22.6	116.5 ± 20.4	<0.001	127.9 ± 22.2	119.2 ± 18.9	0.193	129.8 ± 22.8	116.1 ± 20.7	<0.001	131.3 ± 24.2	117.3 ± 19.7	<0.001

A, atrial; AP, atrial pacing; LEARN, programming mode of the rate response functionality that enables the calibration of the activity sensors; RR, rate response; V, ventricular; VP, ventricular pacing; Z, impedance.

^a^Median [Q1; Q3].

^b^Mean ± standard deviation.

**Figure 1:**
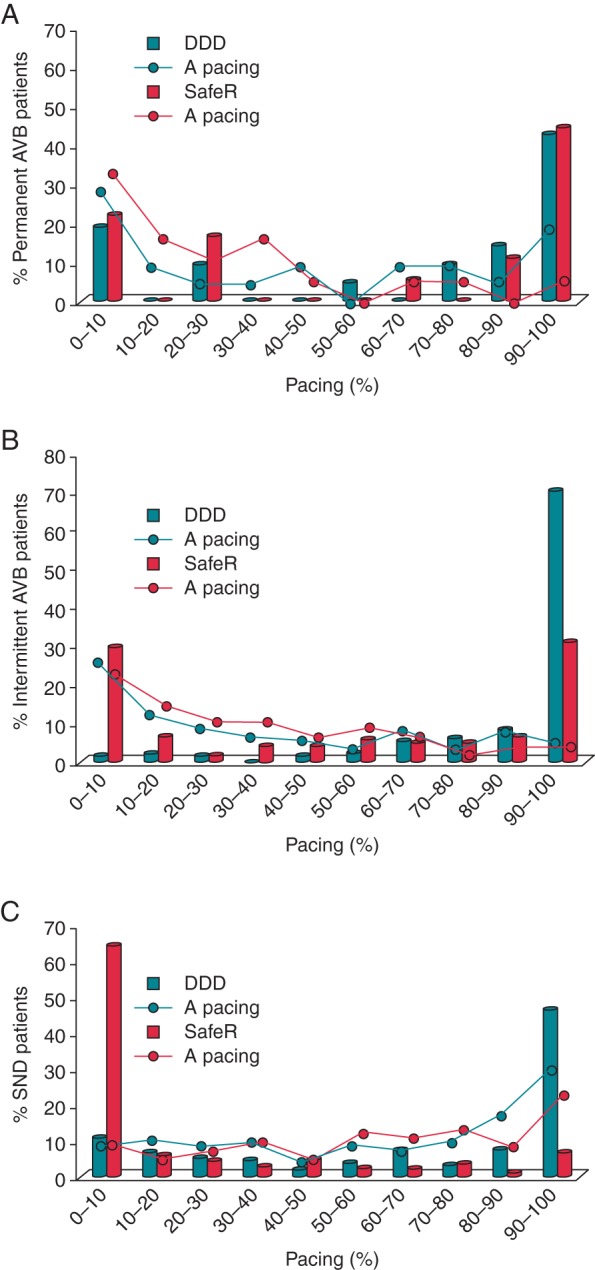
Distribution of VP percentages in patients on SafeR (red bars) vs. DDD pacing (turquoise bars), respectively: (*A*) patients with permanent AVB; (*B*) patients with intermittent AV block; and (*C*) patients with SND.

The distribution of VP in patients with permanent AVB was similar in both treatment arms (*Figure 1A*). In patients with intermittent AVB (*Figure 1B*), SafeR almost completely prevented VP (<10% VP) in more than one-third of patients (29.8%), whereas this low range was achieved only very rarely (1.6%) in the DDD group. Accordingly, 70.5% of patients with intermittent AVB had >90% VP in the DDD group, whereas only 31.4% of patients with intermittent AVB belonged to this high range of pacing percentages in the SafeR group. In SND patients (*Figure 1C*), the vast majority (64.8%) of patients in the SafeR group had <10% VP, whereas only few (11.0%) SND patients had <10% pacing in the DDD group. On the other hand, the applied DDD mode, despite the relatively long nominal AV delays, produced >90% VP in 46.9% of patients, whereas only 6.8% of SND patients had >90% VP in the SafeR group.

### Mortality, system-related and procedure-related adverse events

No differences in the occurrence of death, syncope, or pacing mode intolerance events were observed between groups and implant indications (*Table 3*). None of the syncopal events related to a recorded AVB III episode. The event-related analysis of stored electrograms did not reveal any ventricular arrhythmias prompted by SafeR changeovers.

**Table 3:** Number of patients experiencing death, syncope, or pacing mode intolerance events

	All				AVB all				SND			
	BR (*N* = 650)	SafeR (*N* = 314)	DDD (*N* = 318)	*P-*value^a^	BR (*N* = 310)	SafeR (*N* = 145)	DDD (*N* = 157)	*P-*value	BR (*N* = 336)	SafeR (*N* = 167)	DDD (*N* = 160)	*P-*value^a^
Death, *n* (%)												
All causes	2 (0.3)	26 (8.3)	30 (9.4)	0.610	2 (0.6)	12 (8.3)	15 (9.6)	0.697	0	14 (8.4)	15 (9.4)	0.752
Cardiac death	1 (0.2)	5 (1.6)	11 (3.5)	0.135	1 (0.3)	2 (1.4)	6 (3.8)	0.286	0	3 (1.8)	5 (3.1)	0.494
Syncope, *n* (%)	2 (0.3)	5 (1.6)	9 (2.8)	0.419	1 (0.3)	1 (0.7)	4 (2.5)	0.373	1 (0.3)	4 (2.4)	5 (3.1)	0.746
Pacing mode intolerance, *n* (%)	2 (0.3)	3 (1.0)	1 (0.3)	0.371	1 (0.3)	2 (1.4)	0	0.230	1 (0.3)	1 (0.6)	1 (0.6)	1.000

BR, before randomization; *n* (%), number (%) of patients; AVB, atrioventricular block; SND, sinus node dysfunction.

^a^Statistical test between SafeR and DDD.

Atrial or ventricular lead-related AE occurred in six patients before randomization (0.9%), zero patients in the SafeR group (0%), and five patients in the DDD group (1.6%), respectively. The rate of lead-related AE did not differ between the treatment arms (*P* = n.s.). Pocket haematoma occurred in four patients (0.6%), all before randomization. Pocket infection occurred in seven patients (1.1%), in three patients before randomization, and in four patients after randomization without difference between groups.

Neither device failure nor premature battery depletion was reported.

### Battery impedance at 3-year follow-up and estimated total device longevity

Device settings, including pacing output and width, and rate response, were left to physicians' discretion and are reported in *Table 2*. In these settings, the median [Q1; Q3] device battery impedance at 3-year follow-up was significantly lower in the SafeR (0.45 kΩ [0.30; 0.59]) vs. DDD group (0.53 kΩ [0.34; 0.67], *P* = 0.001). A significantly lower battery impedance was observed between groups in AVB patients (*P* = 0.009; *Table 2*), whereas a trend towards lower battery impedance in SafeR vs. DDD was observed in SND patients (*P* = 0.066). No significant difference was observed in permanent AVB patients (*P* = 0.481), whereas in intermittent AVB patients, the battery impedance was significantly lower in SafeR vs. DDD (*P* = 0.011, *Table 2*).

The mean estimated device longevity was 123.7 ± 22.8 months across the entire population. Prolonged device longevity was calculated for the SafeR group (130.6 ± 23.4 months) vs. the DDD group (117.0 ± 20.0 months), with an MD of 14 months (CI: 10–17 months, *P* < 0.001; *Table 2*). This difference was present in AVB (MD: 13 months, CI: 8–18 months, *P* < 0.001) and SND (MD: 14 months, CI: 9–19 months, *P* < 0.001) patients. No significant difference was observed in the subgroup of permanent AVB patients (MD: 9 months, CI: −5 to 22 months, *P* = 0.193), whereas in intermittent AVB patients, anticipated device longevity was again longer on SafeR vs. DDD (MD: 14 months, CI: 8–19 months, *P* < 0.001, *Table 2*).

### Estimated total device longevity at preset programmed parameters and expected replacements

At fixed pacing amplitudes and pulse widths in both cavities (2.5 V and 0.35 ms, respectively), with rate response OFF and lead impedances approximated to 500 Ω, the median [Q1; Q3] anticipated total device longevity was 130.2 months [115.7–153.3] across the entire population. Increased calculated longevity was obtained for the SafeR vs. the DDD group (142.0 months [124.3; 158.5] and 121.4 months [109.8; 141.6], respectively; *P* < 0.001). In contrast, no impact of the pacing mode on prognosticated device longevity was observed in permanent AVB patients (SafeR: 134.8 months [119.2; 161.2]) and DDD (123.1 months [115.0; 147.8], *P* = 0.237). In intermittent AVB patients, the calculated device longevity differed again significantly in favour of the SafeR group (140.1 months [122.3; 158.2]) vs. the DDD group (120.7 months [110.9; 131.6], *P* < 0.001). Similarly, in SND, the anticipated device longevity was longer with SafeR (142.7 months [129.3; 158.5]) compared with DDD (122.9 months [109.0; 148.8], *P* < 0.001).

**Figure 2:**
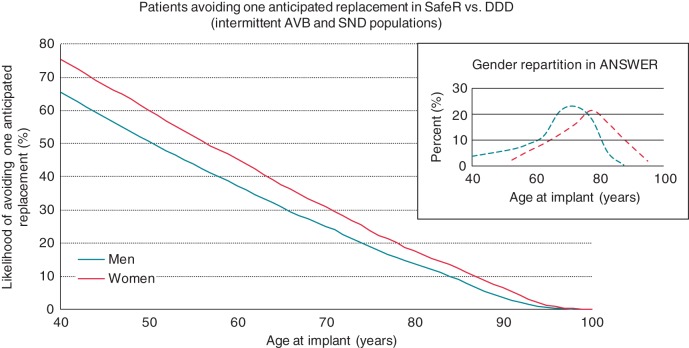
Percentage of intermittent AVB and SND patients avoiding one anticipated replacement on SafeR vs. DDD, according to age at implant (men: turquoise line; women: red line).

According to the applied model, the calculated increase in device longevity on SafeR vs. DDD in both intermittent AVB and SND patients translated into an anticipated reduction of at least one replacement in a considerable percentage of patients. *Figure 2* displays the calculated probability of one prevented device replacement intervention by patient age at implant. On average, 28% of women and 23% of men would avoid one replacement on SafeR across their lifespan when compared with DDD.

## Discussion

The main result of our study is that the SafeR bidirectional AAI–DDD changeover mode for VP minimization can safely and effectively be applied in a general dual-chamber pacemaker population, implanted for intermittent and permanent AVB as well as for SND. We demonstrate that, in SafeR vs. DDD with conservatively programmed AV delays, the decrease in VP was accomplished in intermittent AVB as well as in SND, whereas patients with permanent AVB had similar VP rates on both investigated modes. Ventricular pacing reduction by the SafeR mode has been achieved without compromising safety in both indication subgroups. In addition, VP prevention translated into a significant increase in estimated device longevity and a decrease in the rate of anticipated replacement operations in intermittent AVB and SND patients, while understandably no significant difference resulted in permanent AVB patients.

Close to half of the ANSWER population was implanted for SND, without evidence of AVB at the time of implant. In this group of patients, VP could almost completely be prevented by SafeR, whereas the DDD mode produced a very high percentage of presumably unneeded VP. This effect, achieved through AAI pacing with added VP in case of intermittently impaired AV conduction, is similar to what has previously been reported with a different AAI-DDD changeover mode, the MVP™ algorithm.^5,7^

Importantly, also patients with intermittent AVB experienced a substantial reduction of VP under SafeR. Two previous small studies with short follow-up reported effective VP prevention in patients with AVB through the MVP™ AAI-DDD changeover algorithm.^15^ But the application of the MVP™ mode in patients with AV block has been criticized, because even a marked first-degree AV block does not prompt the addition of VP.^12^ Adverse haemodynamic effects of long PR intervals through pronouncedly prolonged intrinsic AV conduction comprise shortening and impairment of LV filling, pre-systolic mitral regurgitation, and increased left atrial pressure. In some patients, symptoms resembling pacemaker syndrome are provoked by the unfavourable prolongation of the AV electromechanical sequence.^16,17^ Hence, VP prevention in the case of marked first-degree AVB may unintentionally impair overall haemodynamics despite preserving ventricular synchronicity. In view of this possible shortcoming, the more complexly adapting SafeR mode may be regarded as particularly appropriate to achieve low VP percentages in patients with AVB without provoking disadvantageously decoupled AV activation patterns. The results of this sub-analysis confirm the safety and effectiveness of SafeR in intermittent AVB patients.

As expected, patients with permanent AVB did not experience any reduction in VP with SafeR, as this sub-population requires continuous VP. But it is important to note that the investigated changeover mode appeared to be safe and well tolerated even in lasting complete AVB. For this population, no benefit through VP prevention can be anticipated, except where the spontaneous AV conduction is recovering unexpectedly.

The estimated longevity of the devices programmed on DDD approached 10 years, which would already represent a notable improvement compared with many older pulse generators' battery durability. The significant reduction in VP achieved by the SafeR pacing mode when compared with the standard DDD mode translated into an additional calculated gain of >1 year in device longevity. This potential benefit resulted similarly for SND and intermittent AVB patients. A previous study by Benkemoun *et al.*^18^ reported longer estimated longevities on SafeR (142.7 ± 29.9 months) and similar values on DDD (118.8 ± 28.0 months). This discrepancy may, in part, be explained by different programming strategies, in particular the handling of rate response settings. Without an accountable reason, in our study, a high proportion of patients (∼60%) remained programmed in the ‘LEARN’ setting of the rate response algorithm, which enables the algorithm to calibrate the activity sensors according to an individual patient's activity. By this algorithm, the rate response slope and the individualized adaptation of the pacing rate are determined. The frequent and lasting usage of this setting may have contributed to increased battery drain. The analysis of anticipated device longevity at preset programmed parameters and with rate response OFF confirms this hypothesis, with an estimated median longevity in SafeR of 142.0 months. Expectedly, the gain in estimated device longevity observed in patients programmed to SafeR contributed to a decrease in the age-dependent rate of anticipated device replacement on this pacing mode. This is certainly relevant in an elderly population, but even more in younger patients. Taking into account the known risk of infection and the incremental cost of generator replacement surgery,^19^ the possible prolongation of the devices' lifecycle adds an important obvious advantage of the SafeR mode and similar VP prevention algorithms compared with standard DDD pacing. This appears to be particularly important in the light of a recently published study (Clinical Outcome of Pacemaker patients according to pacing Modality and primary INDications: OPTI-MIND) showing considerable sub-optimal programming of dual-chamber pacing in clinical practice.^20^

### Limitations of the study

This study relates to the investigation of a proprietary algorithm by a single device manufacturer. The analysis of VP was based on the device files collected at each follow-up. Not all files were available at the time of the analysis, and the LOCF method was applied to account for individuals with missing outcome data. Another limitation may be the number of programming changes between the arms. However, the very large difference in rates of VP between the SafeR and DDD arms in ANSWER means that these limitations are highly unlikely to have affected the study outcome and there is every reason to believe it reflects a true effect of SafeR vs. DDD.

The device longevity values were predicted values assessed through a device-based prediction algorithm, which may therefore differ from real device longevity. In addition, the rate of replacement was anticipated based on a model taking into account device longevities and survival probabilities that were estimated using a computational approach. This model does not account for those patients who, during the course of time, may have changed their underlying electrical disease (from intermittent to permanent AVB).

## Conclusion

Our study demonstrates that the SafeR pacing mode is effective in reducing the prevalence of VP in a mixed dual-chamber pacemaker population including intermittent and permanent AVB patients. No safety issues were associated with the use of SafeR in both AVB and SND patients over 3 years. The reduction of VP achieved with the SafeR pacing mode translates into increased expected device longevity in intermittent AVB and SND patients, along with a decrease in the rate of anticipated device replacements.

## Supplementary material

Supplementary material is available at *Europace* online.

## Funding

The ANSWER trial has been funded by SORIN CRM SAS.

## Supplementary Material

Supplementary Data
