# Supplementary material for: Safety and efficiency of ventricular pacing prevention with an AAI-DDD changeover mode in patients with sinus node disease or atrioventricular block: impact on battery longevity—a sub-study of the ANSWER trial
Source: Europace. 2015 Nov 26;18(5):739–46. doi: 10.1093/europace/euv358 (PMC5841592; doi:10.1093/europace/euv358)
Supplement: Supplementary Data [file euv358_supplementary_data.zip › euv358supp.docx]

**Appendix**

**Steering committee members:**

PD Dr. Martin STOCKBURGER, MD *(Principal investigator)*

Department of Cardiology and Angiology

Charité University Hospital, Berlin, Germany

Serge BOVEDA, MD

Arrhythmia Department

Clinique Pasteur, Toulouse,, France

Javier MORENO PLANAS, MD

Arrhythmia Department

University Hospital San Carlos, Madrid, Spain

& Arrhythmia Department

Hospital Ramón y Cajal, Madrid, Spain

Pascal DEFAYE, MD

Cardiology Department

A. Michalon University Hospital

Grenoble, France
